# Utilisation of the Scepter Mini dual-lumen balloon – An illustrative series

**DOI:** 10.1177/15910199231216759

**Published:** 2023-11-28

**Authors:** Sean Thomas O’Reilly, Eef Jacobus Hendriks, Ze’ev Itsekson, Rabab Alshahrani, Emily Chung, Ivan Radovanovic, Ronit Agid, Patrick Nicholson, Timo Krings

**Affiliations:** 1 Division of Neuroradiology, Joint Department of Medical Imaging, 156555 Toronto Western Hospital, University Health Network, Toronto, Ontario, Canada; 2 Department of Neuroradiology, 156555 Royal Victoria Hospital, Belfast, County Antrim, UK; 3 Department of Neurosurgery, 26625 Toronto Western Hospital, University Health Network, Toronto, Ontario, Canada

**Keywords:** Arteriovenous malformation, dural arteriovenous fistula, balloon micro catheter, embolisation, cerebral angiography

## Abstract

**Background:**

Dual-lumen balloon microcatheters can aid in the safety and efficacy of endovascular embolisation of cerebrospinal vascular malformations. The Scepter Mini dual-lumen balloon is a novel device with a smaller profile than previous balloon microcatheters, opening up new indications not only in the treatment of cerebrospinal malformations but in various other neurovascular therapeutic and diagnostic scenarios.

**Methods:**

Following institutional ethics review board approval, a retrospective review of our prospectively maintained database of cases employing the Scepter Mini dual-lumen microballoon catheter was conducted. Five cases in particular were highlighted, demonstrating utilisation of this device, which may be of interest to the Neurointerventionalist. Patient demographics, procedure details, complications and clinical outcome data were reviewed.

**Results:**

Five cases employing the Scepter Mini dual-lumen microballoon catheter are presented; trans-arterial embolisation of cerebral AVM, pre-operative tumour embolisation, diagnostic angiography, trans-venous embolisation of cerebral AVM and trans-arterial embolisation of DAVF. No intraprocedural complications were recorded, one patient had a delayed haemorrhage.

**Conclusion:**

Potential utilisation of the Scepter Mini lies not only in the trans-arterial embolisation of cerebrospinal vascular malformations, but in a range of other diagnostic and therapeutic indications as demonstrated.

## Introduction

Endovascular treatment of cerebral arteriovenous malformations (AVMs) and dural arteriovenous fistulas (DAVFs) with the use of liquid embolic agents (LEAs) is long established,^1–4^ alongside other treatment modalities including open surgery and radiosurgery.

A major challenge related to the use of LEAs, in particular ethylene vinyl alcohol (EVOH) copolymers, is proximal reflux within the selected arterial feeder before the embolic agent can penetrate through the nidal vessels of the malformation to reach and completely obliterate the initial venous recipient vessel, necessary to achieve definitive cure. Multiple methods have been described to accomplish nidal penetration, including the use of detachable tip microcatheters, dual microcatheter techniques (such as the ‘pressure cooker technique’) and dual-lumen dimethyl sulphoxide (DMSO) compatible balloons.^5–8^

Each of these techniques represents some disadvantage, which may make them unsuitable in a given arterial anatomy: detachable tip microcatheters allow for prolonged injections but can lead to significant reflux along the parent vessel and thus require a 1.5–5 cm security margin towards a normal feeding vessel. Thus, this technique is often unsuitable for the intracranial pial circulation. The pressure cooker technique requires the insertion of two microcatheters into the same vessel and current DMSO compatible balloons cannot be easily navigated into small or distal branches. Thus, the lack of dual-lumen DMSO compatible balloon microcatheters small enough to navigate into distal vessels, has precluded their use in many AVM/DAVF morphologies.^9^

The Scepter Mini (MicroVention Inc., Aliso Viejo, CA, USA) is a dual-lumen DMSO balloon catheter with a 1.6 F distal diameter and 165 cm working length, which aims to fulfil this niche. Several recently published studies have demonstrated the early experiences working with this new device, and its utilisation in treating cerebrospinal vascular malformations.^10–16^

In this report, we present a single centre case series demonstrating our experiences with the Scepter Mini device, highlighting cases, which exhibit various indications for the device in neurovascular therapeutic and diagnostic scenarios.

### The Scepter Mini

**Figure 1.**
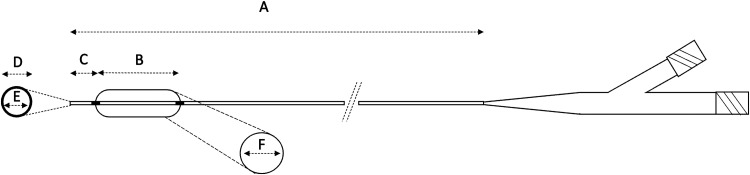
Schematic diagram demonstrating the key features of the Scepter Mini Balloon Microcatheter. Please note radio-opaque markers at either end of the balloon with no distal tip marker. A, working length 165 cm; B, balloon length 9 mm; C, distal tip 2.5 mm; D, distal outer diameter 1.6 F; E, inner diameter 0.010 inches; F, balloon nominal width 2.2 mm at 0.02 ml inflation volume, maximum 2.7 mm at 0.04 ml inflation volume. Proximal diameter 2.8 Fr.

The Scepter Mini (MicroVention Inc., Aliso Viejo, CA, USA) is a dual-lumen DMSO compatible balloon catheter, with a 1.6 F distal outer diameter and 165 cm working length (Figure 1). It features a distal balloon tip, which has a nominal inflation volume of 0.02 ml and 2.2 × 9 mm size (max diameter 2.7 mm at 0.04 ml inflation volume). It is compatible with 0.008-inch microwires or smaller. The balloon features a distal purge hole, covered in a semi-permeable membrane, which in combination with the small inflation volumes requires careful preparation, which is specific to this device and has been described in detail elsewhere.^11^

## Methods

Following institutional ethics review board approval, a retrospective analysis of our prospectively maintained database of cases employing the Scepter Mini device was conducted, between 1 September 2021 and 11 May 2022. In this case series, five different clinical scenarios are highlighted, in which we believe the unique features of the Scepter Mini are of added value for the Neurointerventionalist.

## Results

An overview of included patients baseline clinical data, presentation, useful application of Scepter Mini device, complications and outcome data are presented in Table 1.^17^ All cases used a combination of the Traxcess 7 mini (MicroVention Inc., Aliso Viejo, CA, USA) and Hybrid 007 wires (Balt Neurovascular, France) within the balloon microcatheter.

**Table 1.** Baseline demographic information on included patients, case type and utilisation of Scepter Mini, complications and clinical outcomes.

Case number	Presenting condition	Pre-mRS^17^	Case type	Scepter Mini		Complications		Post-mRS^17^	Follow-up	
				Utilisation	Benefit	Procedural	Delayed		Time (months)	Outcome
1	Parietal AVM	0	Therapeutic	Proximal Flow Control	Prevents unwanted antegrade flow of LEA into draining vein in fistulous type malformation	Nil	Small ICH day 2	0	3	Complete Cure
2	Orbital Tumour	0	Therapeutic	Reflux Protection and antegrade push	Protect origin of CRA and allow penetration of tumour vessels	Nil	Nil	0	NA	Complete Surgical Resection
3	Metameric Spinal AVM	3	Diagnostic	Negating AVM ‘sump-effect’	Aids visualisation of small vessels supplying cord, improving safety of future treatment	Nil	Nil	3	NA	NA
4	Hippocampal AVM	0	Therapeutic	Temporary Venous Plug	Allows trans-venous LEA push through nidus, without first permanently occluding outflow of AVM-reducing risk of bleeding	Nil	Nil	0	3	Complete Cure
5	DAVF	0	Therapeutic	Antegrade Push of LEA	Allows LEA to penetrate through DAVF into induced pial supply, reducing risk of bleeding on occlusion of fistula	Nil	Nil	0	3	Complete Cure

mRS, modified Rankin Scale; LEA, liquid embolic agent; CRA, central retinal artery.

### Case 1 – trans-arterial embolisation of brain AVM

**Figure 2.**
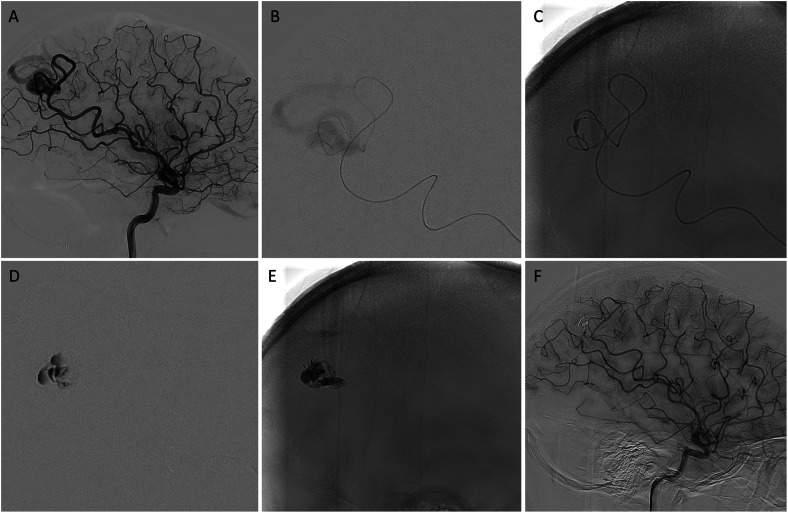
Case 1. Trans-arterial embolisation of brain AVM. Lateral projection DSA of the left ICA shows a small AVM centred in the parieto-occipital region, with superficial venous drainage (A). Microcatheter injection shows the fistulous nature of the AVM, with rapid washout of contrast (B). Proximal flow arrest following Scepter Mini balloon inflation (C) prior to repeat microcatheter angiography (D) shows reduced ‘sump-effect’ of the fistula, with contrast now stagnating within the AVM nidus. Subsequent onyx injection under flow arrest was able to occlude the nidal vessels and proximal vein (E), with follow-up DSA at 3 months showing complete occlusion of the AVM (F).

Patient with a small left parietal AVM discovered on imaging following seizure (Spetzler-Martin grade 2).^18^ Angiography demonstrated a fistulous AVM with two arterial feeders, the left middle cerebral artery (MCA) angular branch and left anterior cerebral artery (ACA) pericallosal branch (Figure 2) which was filling largely from the right internal carotid artery (ICA) across the anterior communicating artery (ACOM). Bilateral common femoral access with 6 Fr Envoy guide catheters (Cerenovus, Irvine, CA, USA) were employed, to allow right sided control angiography.

Use of the Scepter Mini allowed proximal flow control; with formation of a ‘removable’ pressure cooker within the arterial pedicle close to the fistula, avoiding excess distal migration of embolic material prior to closure of the fistula.

Patient had a small intracranial haemorrhage day 2 post-procedure. No new neurological deficit following treatment, and follow-up digital subtraction angiography (DSA) at 3 months showed complete obliteration of the AVM.

### Case 2 – pre-operative embolisation of hypervascular orbital mass

**Figure 3.**
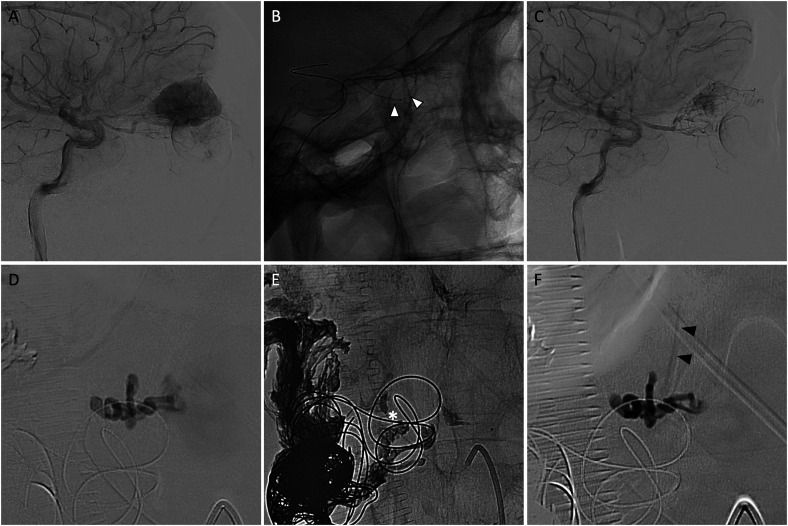
Case 2 (A–C). Pre-operative embolisation of hypervascular orbital mass. Lateral projection DSA centred on the orbit reveals a hypervascular lesion superiorly fed by distal branches of the ophthalmic artery (A). A Scepter Mini microcatheter was able to be navigated in the ophthalmic artery past the origin of the CRA (white arrowheads in B), allowing LEA injection with proximal protection of the CRA origin. Near-complete tumour embolisation was achieved with preservation of the choroidal blush (C). Case 3 (D–F). Embolisation of spinal AVM. Microcatheter injection in a patient with a large paraspinal AVM demonstrates an arteriovenous shunt which may be amenable to LEA embolisation (D). Subsequent inflation of the Scepter Mini balloon (white asterisk in E) and microcatheter run with flow arrest reveals the posterior spinal artery supply arising from this segmental level, precluding safe injection of liquid embolic (black arrowheads in F).

Patient with orbital proptosis secondary to a hypervascular orbital mass, surgical excision with pre-operative endovascular embolisation was planned (Figure 3). Angiography demonstrated supply via branches of the ophthalmic artery distal to the origin of the central retinal artery (CRA). Right radial access was chosen, using a RIST 079 (Medtronic, Dublin, Ireland) guide catheter.

Scepter mini was able to navigate into the ophthalmic over a Traxcess mini 7 wire, however advancement distally was difficult as the proximal portion of the catheter kept prolapsing into the ICA. This was overcome with short inflations of a Scepter C balloon (MicroVention Inc., Aliso Viejo, CA, USA) within the ICA, providing the proximal support necessary to allow navigation of the Scepter mini distal to the origin of the CRA. Use of the Scepter mini allowed anterograde push of liquid embolic into the lesion whilst preventing reflux to the CRA, resulting in near-complete devascularisation with preservation of the choroidal blush. Patient underwent complete surgical removal on day 2 post-embolisation, histology revealed a solitary fibrous tumour.

### Case 3 – embolisation of spinal AVM

Patient with a previously partially treated large metameric paraspinal AVM, presented with worsening myelopathic symptoms related to epidural venous congestion secondary to high-flow shunts. It was decided to proceed with endovascular embolisation of the fistulous components of this paraspinal AVM to reduce congestion from the enlarged epidural venous pouch (Figure 3), please note liquid embolic cast and coils from previous embolisation procedures. A 5 Fr Cobra 038 guide catheter (Merit Medical, Salt Lake City, UT, USA) was used to allow access into the segmental arteries.

Initial angiography demonstrated a portion of the AVM, which was felt to be amenable to embolisation, however a subsequent microcatheter run performed with balloon inflation to reduce the ‘sump-effect’ of the AVM, demonstrated posterior spinal artery contribution arising from this segmental level, thereby precluding injection of embolic material.

### Case 4 – trans-venous embolisation of brain AVM

**Figure 4.**
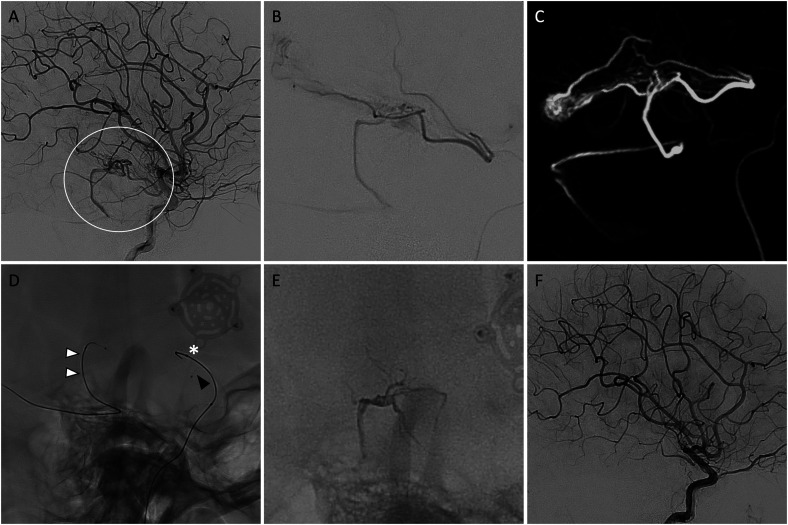
Case 4. Trans-venous embolisation of brain AVM. Lateral projection DSA reveals residual hippocampal AVM with en-passage supply from the anterior choroidal artery and early venous drainage into the hippocampal vein (white circle in A). Microcatheter angiography (B) and subsequent 3D spin (C) from the anterior choroidal artery show the AVM to be supplied by branches arising close to the choroidal point, thereby precluding safe trans-arterial embolisation. Scepter Mini microcatheter was able to be navigated trans-venously to the nidus via the superior petrosal sinus and hippocampal vein (white arrowheads in D). Here we also see the Scepter C balloon (white asterisk) within the right ICA and Magic 1.2 FM (black arrowhead) in the anterior choroidal for control angiography. With arterial flow arrest via inflation of the Scepter C and distal venous plug using the Scepter Mini, LEA was injected through the Scepter Mini and seen to penetrate retrogradely through the nidus into the proximal aspect of the arterial feeders (E). Final control DSA demonstrates complete obliteration of the AVM, with preservation of anterior choroidal artery (F).

Patient with a ruptured right hippocampal AVM. Initial angiography revealed en-passage arterial supply via the anterior choroidal artery close to the choroidal point, and following multidisciplinary board discussion, the patient underwent open neurosurgical resection. Follow-up DSA revealed a small residual arteriovenous shunt and given the previous rupture and surgical procedure, endovascular embolisation was now recommended (Figure 4).

Trans-venous retreatment was preferred given the en-passage supply from the anterior choroidal artery. Trans-venous access was obtained via an 8 Fr groin sheath and Cerebase DA Guide catheter (Cerenovus, Irvine, CA, USA) placed within the contralateral left internal jugular vein, to allow microcatheter access into the right superior petrosal sinus. Trans-arterial access was obtained using NeuronMax 088 (Penumbra, Alameda, CA, USA).

Scepter Mini allowed a temporary retrograde trans-venous ‘pressure cooker’ technique, blocking the draining vein to allow retrograde penetration of liquid embolic from the vein, through the nidus and into the proximal aspect of the arterial feeders. A Scepter C was placed in the ICA to reduce inflow into the anterior choroidal artery and a Magic 1.2 FM (Balt Neurovascular, France) placed in the anterior choroidal to allow for control angiography during the procedure. The patient was discharged without new neurological deficit after 24 hours.

### Case 5 – trans-arterial embolisation of DAVF

**Figure 5.**
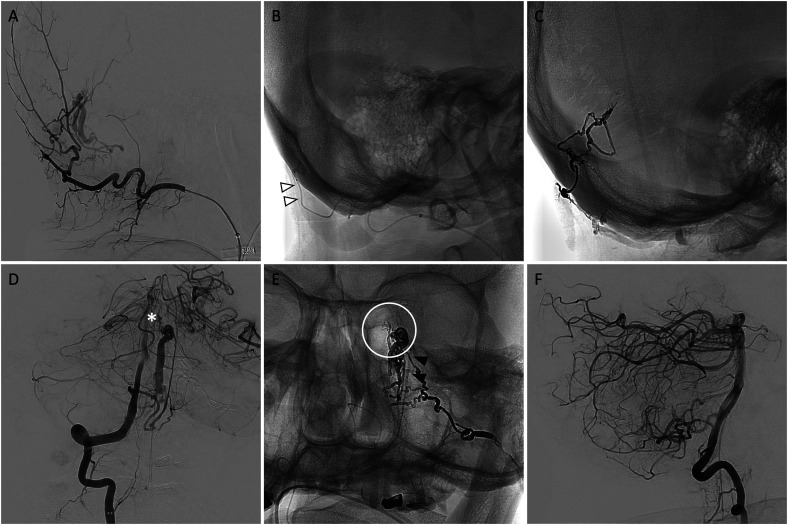
Case 5. Trans-arterial embolisation of DAVF. Lateral projection DSA of the left occipital artery shows a high grade falcotentorial DAVF with cortical venous drainage (A). Scepter Mini microcatheter was navigated into an osteodural feeder, with the catheter tip within the diploic space (white arrowheads in B). Subsequent LEA injection was able to penetrate from the osteodural feeder, through the fistula into proximal pial feeders (C). AP DSA showing residual fistula filling from the posterior meningeal branch arising from the right PICA, with induced pial supply from left SCA (white asterisk in D). A second Scepter Mini was navigated through this branch to the fistulous point (black arrowheads in E), and subsequent LEA injection was able to penetrate into the pial induced supply (white circle in E) reducing risk of haemorrhage and resulting in complete obliteration of the fistula (F).

Patient with an incidentally discovered Borden type 3 falcotentorial DAVF, supplied by osteodural feeders from the left occipital artery and left posterior meningeal artery arising from the right PICA (Figure 5).^19^ There was also induced pial supply from the distal superior cerebellar artery (SCA). Arterial access was via a 6 Fr sheath in the right radial artery, and a Benchmark 071 guide catheter (Penumbra, Alameda, CA, USA).

Scepter Mini placed in the left OA to push liquid embolic through the osteodural feeders to occlude the fistula, with excellent navigability able to track distally into the diploic space. Onyx injection in this branch was terminated when it appeared to penetrate through the perceived point of fistulisation into the first segment of the vein, however subsequent control angiography confirmed incomplete obliteration of the fistula. A second Scepter Mini was then placed distally within the posterior meningeal branch. With balloon inflation we were able to inject LEA not just through the recipient vein, but reflux from there into the induced distal pial vessels also supplying the fistula, thereby preventing haemorrhagic complication.^20^ This resulted in complete cure of the fistula, and the patient was discharged at neurological baseline the next day.

## Discussion

Endovascular embolisation of cerebrospinal vascular malformations with LEAs is well established, with previous studies also demonstrating how the utilisation of dual-lumen balloon catheters can help improve complete embolisation rates and reduce procedure times.^7,8^ There are also a number of studies demonstrating early experience with the Scepter Mini and how it can broaden the application of dual-lumen balloon microcatheters given its smaller size and improved trackability.^10–16^ To this end, we outline several scenarios, which demonstrate different utilisations of the Scepter Mini device.

In Case 1 of a fistulous AVM, we demonstrate how the Scepter Mini balloon not only plugged the arterial feeder (thus reducing unwanted proximal reflux of LEA), but also allowed for proximal flow control (as demonstrated in Figure 2B–D). Thus, we were able to inject LEA in a more controlled manner filling the AVM nidus and proximal draining vein, without excessive distal penetration into the draining vein or unwanted distal venous migration. Given the close proximity to the Rolandic area, the Scepter Mini was preferred to a detachable tip microcatheter as we could then completely remove any catheter material from the eloquent arterial supply following embolisation.

Case 2 demonstrates both the trackability of the Scepter Mini as well as its potential application in both providing a plug to prevent proximal LEA reflux (thus protecting the origin of the CRA), as well as allowing distal push of LEA into the feeding arteries of this hypervascular tumour lesion. In situations such as this with a relatively short reflux margin of safety, use of a dual-lumen balloon may allow an appropriate balance between effective embolisation and reduced risk of complication.

Case 3 illustrates the usefulness of proximal flow control by inflation of the Scepter Mini balloon as an important diagnostic tool, allowing for delineation of small vessels in close proximity to a high-flow region such as in this spinal AVM. Initial microcatheter angiography appeared to show a suitable target for LEA embolisation (Figure 3D–F), however a subsequent run with balloon inflation demonstrated the previously unseen posterior spinal artery supply. The ability to reduce arterial inflow, and so negating the ‘sump-effect’ of high-flow lesions, can allow visualisation of these small vessels, thereby improving safety of endovascular treatment in these types of lesions.

Case 4 again demonstrates the trackability of the Scepter Mini, allowing navigation from the transverse sinus, via the superior petrosal sinus into the hippocampal vein and close to the nidus of this hippocampal AVM. It also shows how utilisation of a dual-lumen balloon in the trans-venous treatment of AVMs can allow for controlled occlusion of the draining vein, thereby allowing LEA to penetrate from the venous side, through the nidus, into the proximal arterial feeders. In this case a Scepter C balloon was also placed in the ICA to reduce arterial inflow to the anterior choroidal, and thus reduce the pressure opposing retrograde penetration of the LEA through the nidus from the venous side.

In case 5, the trackability of the Scepter Mini is again highlighted as well as its ability to provide a robust ‘pressure cooker’, with LEA able to penetrate from the dural branch through the recipient vein, into the induced distal pial supply, thereby preventing haemorrhagic complication on occlusion of the fistula.^20^

Several studies have described complications arising from use of the Scepter Mini, including balloon over-inflation and subsequent vessel rupture.^8^ We have not encountered this in our experience to date, which we feel may be due to our preference for maintaining balloon inflation using a rotating one-way flow valve, rather than a mechanical ‘clickable’ flow switch. This may avoid the sudden small increase in pressure within the balloon inflation port, which could contribute to over-inflation in a system requiring such small inflation volumes. Whilst we cannot confirm which balloon port locking method other centres have employed, it is important for operators to be aware of this potential issue and take steps to help avoid balloon over-inflation.

Another complication reported is proximal balloon migration or so-called balloon ‘jump back’, seen whenever the LEA cast reaches the balloon and the pressure within the injected pedicle exceeds that of the radial occlusive pressure of the balloon, causing it to be pushed proximally.^10^ This may be exacerbated by inadequate anchoring of the balloon and has been seen once in our experience, during embolisation of a DAVF via a superficial temporal artery (STA) branch, which was approximately 2 mm in size. Therefore, care should be taken when using this device in vessels, which are approaching the upper limit in size of the nominal width of the balloon (i.e., vessels larger than 2 mm in diameter), and operators should avoid the temptation to over-inflate balloon to prevent ‘jump back’, due to risk of rupture.^12^

### Limitations

The main aim of this study is to highlight differing clinical scenarios in which the unique features of the Scepter Mini may be of benefit to the Neurointerventionalist, in the hope of improving procedural safety and efficacy. We have not sought to present an exhaustive demonstration of our early experience with this device, as has been presented elsewhere in the literature.

Given the narrow focus of this case series, it is likely prone to bias in terms of case selection. Also, the small sample size of this study may limit interpretation of presented findings.

## Conclusion

The Scepter Mini dual-lumen microcatheter balloon is a promising new tool in the Neurointervenionalist armory. As we have demonstrated, its unique features allow for potential utilisation in multiple diagnostic and therapeutic scenarios.

## Abbreviations

ACA: Anterior cerebral artery
AVM: Arteriovenous malformation
CRA: Central retinal artery
DAVF: Dural arteriovenous fistula
DMSO: Dimethyl sulphoxide
DSA: Digital subtraction angiography
EVOH: Ethylene vinyl alcohol
ICA: Internal carotid artery
ICH: Intracranial haemorrhage
LEA: Liquid embolic agent
MCA: Middle cerebral artery
mRS: Modified Rankin scale
OA: Occipital artery
STA: Superficial temporal artery
